# Specific growth suppression of human cancer cells by targeted delivery of *Dictyostelium* mitochondrial ribosomal protein S4

**DOI:** 10.1186/1475-2867-14-56

**Published:** 2014-06-20

**Authors:** Junji Chida, Hikaru Araki, Yasuo Maeda

**Affiliations:** 1 Division of Molecular Neurobiology, Institute for Enzyme Research, The University of Tokushima, Kuramoto-cho, Tokushima 770-8503, Japan; 2 Division of Enzyme Chemistry, Institute for Enzyme Research, The University of Tokushima, Kuramoto-cho, Tokushima 770-8503, Japan; 3 Department of Developmental Biology and Neurosciences, Graduate School of Life Sciences, Tohoku University, Aoba, Sendai 980-8578, Japan

**Keywords:** Mitochondrial ribosomal protein S4 (MRP4), *Dd-mrp4*, Anticancer action, Apoptosis, Proliferation, Differentiation, Human tumor, *Dictyostelium discoideum*

## Abstract

**Background:**

In general, growth and differentiation are mutually exclusive but are cooperatively regulated throughout development. Thus, the process of a cell’s switching from growth to differentiation is of great importance not only for the development of organisms but also for malignant transformation, in which this process is reversed. We have previously demonstrated using a *Dictyostelium* model system that the *Dictyostelium* mitochondrial ribosomal protein S4 (*Dd-mrp4*) gene expression is essential for the initiation of cell differentiation: *Dd-mrp4*-null cells fail to initiate differentiation, while the initial step of cell differentiation and the subsequent morphogenesis are markedly enhanced in *mrp4*^OE^ cells overexpressing the *Dd-mrp4* in the extramitochondrial cytoplasm. This raised a possibility that the ectopically enforced expression of the *Dd-mrp4* in human cells might inhibit their growth, particularly of malignant tumor cells, by inducing cell differentiation.

**Methods:**

Four kinds of human tumor cell lines were transfected by three kind of vector constructs (the empty vector: pcDNA3.1 (Mock); pcDNA3.1-*rps4* bearing *Dictyostelium* cytoplasmic ribosomal protein S4; pcDNA3.1-*mrp4* bearing *Dictyostelium* mitochondrial ribosomal protein S4). As controls, four kinds of human primary cultured cells were similarly transfected by the above vector constructs. After transfection, growth kinetics of cells was analyzed using cell viability assay, and also the TUNEL method was used for evaluation of apoptotic cells.

**Results:**

Ectopically expressed *Dd-mrp4* suppressed cell proliferation through inducing apoptotic cell death specifically in the human lung adenocarcinoma (A549), epithelial cervical cancer (HeLa), hepatocellular carcinoma (HepG2) and colonic carcinoma (Caco-2), but not in primary cultured normal cells, such as human brain microvascular endothelial cells (HBMECs); human umbilical vein endothelial cells (HUVECs) and human normal hepatocytes (hHeps™), with one exception (human cardiac fibloblasts (HCF)).

**Conclusion:**

The present finding that the ectopically enforced expression of *Dd-mrp4* in human several tumor cell lines specifically suppresses their proliferation suggests strongly that the *Dd-mrp4* gene derived from *Dictyostelium* mitochondria may provide a new promising therapeutic strategy for disrupting cell viability pathways in human cancers.

## Background

Apoptosis is a process of cell death that serves as a major mechanism for precise regulation of cell numbers, and as a defense mechanism to remove potentially dangerous cells like tumor cells. Apoptosis also covers important functions in a wide range of cellular processes ranging from growth to differentiation. Mitochondria exert a key role in many pathways leading to programmed cell death [1-3], though the precise mechanisms underlying the role in apoptosis remain to be elucidated [4-7]. Although regulation of the cell death machinery has been shown to be somewhat different from one species to another, from studies in *Caenorhabditis elegans*, *Drosophila melanogaster* and mammals, it is mainly controlled by mitochondrial proteins. In mammals, activation of caspases (cysteine proteases that are the main performer of apoptosis) is under the tight control of the Bcl-2 family proteins that primarily act by regulating the release of caspase activators from mitochondria as the central administrator of apoptosis [8,9].

The mammalian mitochondrial ribosome (mitosome) is largely responsible for the synthesis of 13 proteins of the inner membrane, and these proteins are components of the oligomeric complexes essential for oxidative phosphorylation [10,11]. Accordingly, mitosomes synthesise a substantial amount of cellular components needed to generate ca. 90% of the ATP required for eukaryotic cells. Some studies have identified several mitochondrial ribosomal proteins as apoptosis-inducing factors, including the death-associated proteins DAP3 and PDCD9 [2,12]. Mitochondrial ribosomal protein L41 (MRPL41) suppresses the growth of cancer cells in nude mice, by induction of p53-induced mitochondrion-dependent apoptosis [13]. Saini et al. [14] have also demonstrated that S29 ribosomal protein (RPS29) induces mitochondria-mediated apoptosis of the human laryngeal carcinoma cell line (Hep2 cells) through the activation of p38 MAPK and JNK signaling. Recently, Tsofack et al. [15] have immunohistochemically revealed that high expression of the X-linked ribosomal protein S4 (RPS4X; encoded by human sex-chromosome X), which is involved in cellular translation and proliferation, is implicated for less aggressive ovarian tumors, slower disease progression, and less deaths associated with this disease, while that lower levels of RPS4X are correlated to poor survival and disease progression.

Based on numerous genome analyses, mitochondria are believed to be originated from an early endosymbiotic event between a eubacterium and its host cell, and the closest free-living relatives of mitochondria are suggested to be members of the rickettsial subdivision of the α-proteobacteria. Therefore, the mitochondrial ribosomal protein (MRP) has been generally expected to display higher structural and functional similarities to a bacterial ribosome than to a eukaryotic cytoplasmic ribosome.

*Dictyostelium discoideum* is a social amoeba whose life cycle consists of two distinct phases—growth and differentiation—that are easily controlled by nutritional conditions. *D. discoideum* (axenic strain Ax-2, Ax-3, or Ax-4) cells grow and multiply by mitosis as long as nutrients are supplied. Upon exhaustion of nutrients, however, starving cells initiate to differentiate and aggregate each other by chemotaxis to form multicellular structures. The cell aggregate (slug) eventually culminates to form a fruiting body consisting of a mass of spores (sorus) and a supporting cellular stalk. The life cycle of *Dictyostelium* cells is relatively simple, but it contains almost all of the cellular processes (movement, adhesiveness, differentiation, pattern formation, *etc*.) essential for the establishment of multicellular organization. In basically haploid *Dictyostelium* cells, gene disruptions by homologous recombination are available for analysis of precise gene functions. Insertional mutagenesis by the restriction enzyme–mediated integration (REMI) method has also been established to isolate and characterize intriguing functional genes [16]. Thus *Dictyostelium* is a useful model system for analyzing a various aspects of cellular development. The process of a cell’s switching from growth to differentiation is of great importance not only for the development of organisms but also for malignant transformation, in which this process is reversed. Using axenic strain Ax-2 cells, we have precisely specified a critical checkpoint (growth/differentiation transition or GDT point), from which cells start differentiation in response to starvation, in the cell cycle of *Dictyostelium* cells [17-19]. Accordingly, integration of GDT point–specific events with starvation-induced events is needed to understand the mechanism regulating GDTs. Beyond our imagination, increasing evidence indicates that mitochondria have novel, essential, and multiple functions as the regulatory machinery of the initiation of differentiation, cell-type determination, cell movement and pattern formation [20]. For example, a mitochondrial gene cluster (*dia3* consisting of *nad11*, *nad5*, *rps4*, *rps2*, and *nad4L*) including ribosomal protein S4 (*mrp4* of *Dictyostelium discoideum* cells: *Dd-mrp4*), are specifically expressed in response to starvation around the GDT point and play essential roles in the initiation of cell differentiation in Ax-2 cells [21]. Partial disruption of *Dd-mrp4* by homologous recombination causes impaired differentiation, thus resulting in the failure of many cells to aggregate [21]. Transformants (*Dd*-*mrp4*^AS^ cells) generated by antisense-mediated gene inactivation also exhibit markedly delayed differentiation [21]. Moreover, *mrp4*-null cells created by an elegant method, in which only the *mrp4* gene was specifically disrupted by a combination of homologous recombination and delivery of an appropriate restriction endonuclease (*Sfo*I) into mitochondria, fail to differentiate even after a prolonged time of starvation [20,22]. In contrast, *Dd*-*mrp4*^OE^ cells ectopically overexpressing *Dd-mrp4* exhibit markedly enhanced differentiation after starvation [21]. Taken together these data raised a possibility that overexpression of the extraneous *Dd-mrp4* gene in human cells might inhibit their proliferation, particularly of malignant tumor cells, by inducing terminal differentiation including programmed cell death (apoptosis). To test this possibility, effects of enforced expression of the *Dd-mrp4* on the proliferative activity of several lines of tumor and primary cells were examined in the present work, using cell viability assay and the TUNEL method. As was expected, our results have demonstrated that the ectopically enforced *Dd-mrp4* expression specifically suppresses proliferation of all the human tumor cell lines examined, by inducing cell differentiation which is possibly attributable to either the attenuated pro-apoptosis signaling. This finding strongly suggested that the *Dd-mrp4* mRNA and/or its product (Dd-MRP4 protein) might act as a new promising target for specifically disrupting cell viability pathways in human tumor cells and consequently for cancer therapy.

## Methods

### Cell lines and cell culture

HBMECs (human brain microvascular endothelial cells) and HUVECs (human umbilical vein endothelial cells) were purchased from DS Pharma Biomedical (Osaka, Japan). HBMECs and HUVECs were cultured in collagen-coated plates in HuMedia EG-2 (Kurabo, Osaka, Japan). HCF (Human cardiac fibroblasts) were purchased from ScienCell Research Laboratories (Carlsbad, CA). HCF were cultured in RPMI 1640 medium with L-glutamine and 10% FBS. hNHeps™ (Human normal hepatocytes) cells were purchased from Lonza (Lonza, Walkersville, MD). hNHeps™ cells were maintained in hepatocyte culture medium (Lonza). A549 (human lung adenocarcinoma) and HeLa (human epithelial cervical cancer) cells were purchased from Sigma-Aldrich (St. Louis, MO). HepG2 (human hepatocellular carcinoma) cells were provided from Cosmobio (Tokyo, Japan). Caco-2 (human colonic carcinoma) cells were purchased from American Type Culture Collection (Rockville, MD). A549, HeLa, HepG2, and Caco-2 cells were maintained in Dulbecco’s modified Eagle’s medium (DMEM) (Wako Pure Chemical Industries, Osaka, Japan) containing 50 units/ml penicillin and 50 μg/ml streptomycin (Sigma-Aldrich, St. Louis, MO), and supplemented with 10% FBS (Wako Pure Chemical Industries). These cells were cultured at 37°C in a humidified atmosphere of 5% CO_2_.

### Construction of the expression vector of the *rps4* gene and *mrp4* gene

**Figure 1:**
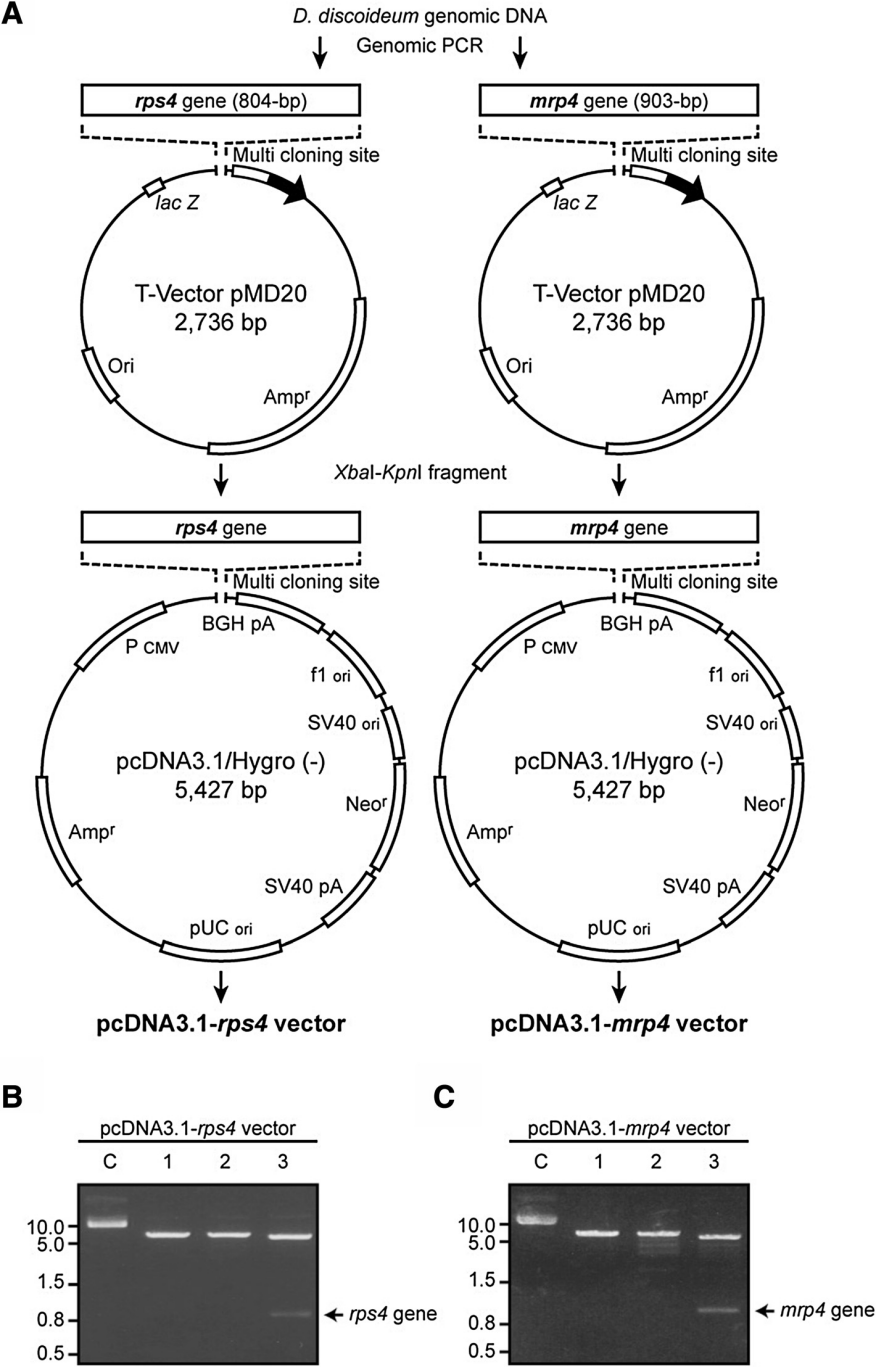
**Construction of expression vectors. (A)** A schematic representation of the recombinant plasmids. The pcDNA3.1-*rps4* vector was constructed by inserting the *rps4* gene fragment into *Xba*I and *Kpn*I sites of pcDNA3.1/Hygro (-) between the P_CMV_ (cytomegalovirus promoter) sequence and the BGHpA (bovine growth hormone polyadenylation) sequence. The pcDNA3.1-*mrp4* vector was constructed by inserting the *Dd-mrp4* gene into pcDNA3.1/Hygro (-), in the same way as pcDNA3.1-*rps4* vector. **(B)** Identification of the pcDNA3.1-*rps4* vector by using restriction enzyme digestion and agarose gel electrophoresis. Lane C, non-digested; lanes 1-3, expression vector digested with *Xba*I, *Kpn*I, and *Xba*I &*Kpn*I, respectively. **(C)** Identification of the pcDNA3.1-*mrp4* vector by using restriction enzyme digestion and agarose gel electrophoresis. The lanes are the same as those shown in **(B)**.

Genomic DNAs were extracted from *D. discoideum* (strain Ax-3) cells according to the methods described previously [23]. The DNA fragments that encode the full-length *rps4* gene (804-bp) or *mrp4* gene (903-bp) were separately amplified from the genomic DNA by PCR using the following amplification conditions; a 2 min pre-denaturing step at 94°C followed by 35 cycles of amplification, with a 10-denaturing step at 98°C, a 30- sec annealing step at 55°C, and a 1-min extension step at 72°C. The final extension step was 10 min at 72°C. The primers used are listed in Table 1. The PCR-amplified *rps4* gene and *mrp4* gene were first cloned into the pMD20-T vector (Takara Bio, Otsu, Japan) and then cloned into the pcDNA3.1/Hygro (-) vector to construct the pcDNA3.1-*rps4* vector and pcDNA3.1-*mrp4* vector, respectively (see Figure 1). To estimate microscopically the efficiency of vector transfection, a vector contrast (pcDNA3.1-*gfp*) bearing the full-length *gfp* gene (772 bp) instead of the full length *rps4* or *mrp4* gene was also prepared.

**Table 1:** Oligonucleotide primers used in this study

**Primer name**	**5′-3′ nucleotide sequence**
NS4-FH3	gatggatccatggctcgtggtccaaa
NS4-RB1	gcaagcttttaagcaacggtttcgattttttcacc
MtS4-FH3	taggatccatgagacaacgaaaaaatgtgacaaaattt
MtS4-RB1	cgaagcttttatcttagtcttttatatttctttaataaag

### Transfection

A single day prior to transfection, cells were plated in 6-well culture dishes at a density of 5.0 × 10^5^ cells per well (2.0 ml/well). Transfection was performed using Lipofectamine 2000 reagent (Invitrogen, Carlsbad, CA) with methods as recommended by the manufacturer. In brief, transfection was initiated when the cells were 70-80% confluent. For each well, 4 μg plasmid DNA was added into 250 μl of Opti-MEM (Invitrogen), 5 μl of lipofectamine 2000 into 250 μl of Opti-MEM, and then mixed plasmid DNA with Lipofectamine 2000. The mixture was added to cells in the 6-well plates, giving an end volume of 1 ml. The Opti-MEM medium containing the complexes was incubated for 6 hrs at 37°C, then replaced with 2 ml of standard growth media and cultured at 37°C for 42 or 66 hrs. Two days after transfection, cell viability was measured as described below.

### Cell viability measurements

Cell viability was determined using cell counting kit-8 (Dojindo, Kumamoto, Japan) according to the manufacture’s instructions. Briefly, the day before cell counting, trypsinized cells (2 × 10^4^ cells/well) were plated in 96-well plates, and 16 hrs later, incubated in 96-well plates with a tetrazolium compound (WST-8) solution (10 μl/well) at 37°C for 2 hrs. The quantity of formazon product measured at 450 nm is directly proportional to the number of live cells in the culture. The number of viable cells was assessed by measurement of absorbance at 450 nm using Multiskan JX (Thermo electron corp., Madison, WI). The experiments were repeated in triplicate wells.

### TUNEL assay

A TUNEL (terminal deoxynucleotidyl transferase dUTP nick end labeling) assay, a common method for detecting apoptotic programed cell death (DNA fragmentation that results from apoptotic signal cascades) was carried out using the *In Situ* Cell Death Detection Kits (Roche Diagnotics Corp.) according to the manufacturer’s instructions. Briefly, transiently transfected HepG2 cells were grown on chamber slides. After 48 hrs, cells were fixed immediately in 4% (vol/vol) paraformaldehyde for 1 hr, permeabilized using 0.3% Triton X-100, and then incubated at 37°C for 1 hr with TdT-mediated TUNEL reaction mixture containing FITC (fluorescein isothiocyanate)-conjugated anti-Br-dUTP mAb (monoclonal antibody). For a positive control, HepG2 cells were treated with DNase I as specified by the manufacturer.

### Statistical analysis

All the data were expressed as mean ± SD. The statistical significance of difference in cell viability assay was analyzed using the Student’s t-test. Results represent data from three independent experiments for each group, and *P*-values of <0.05 were considered statistically significant.

## Results

### Similarities of RPS4 and MRP4 in the amino acid sequences between human and *Dictyostelium*

Mitochondrial ribosomes (MRPs) contain bacteria-type proteins reflecting their endosymbiotic heritage. After reflection on the matter, a subset of these genes is retained within the mitochondrion in eukaryotic cells, but most of mammalian MRPs are products of nuclear genes. Thus these proteins are synthesized in cytoplasmic ribosomes by mitochondria for assembly with the mitochondrially encoded rRNA [24]. Unexpectedly, mammalian MRPs (55S) are different from bacterial (70S) and cytoplasmic ribosomes (80S), as well as other kinds of mitochondrial ribosomes. For example, human MRPs are devoid of several of the major RNA stem structure of bacterial ribosomes but they hold a higher number of proteins (ca. 80 proteins), suggesting a model where proteins may displace RNA structural elements during the evolution of these ribosomes [25]. Thus MRPs are imported into mitochondria where they assemble cooperatively with mitochondrially transcribed rRNAs into ribosomes that are responsible for translating the 13 mRNAs for essential proteins of the oxidative phosphorylation system.

To monitor the effect of ectopically enforced Dd-MRP4 expression on growth kinetics of human cells, Dd-MRP4-expressing human cells (*Dd-mrp4*^OE^-cells) and Dd-RPS4-expressing human cells (*Dd-rps4*^OE^-cells as a negative control) were prepared by transfection using the pcDNA3.1-*mrp4* vector and pcDNA3.1-*rps4* vector, respectively. Several human primary cell lines such as brain microvascular endothelial cells (HBMECs) and umbilical vein endothelial cells cardiac fibroblasts (HUVECs) were also transformed using the pcDNA3.1 (Mock), pcDNA3.1-*rps4* or pcDNA3.1-*mrp4* vector as controls.

**Figure 2:**
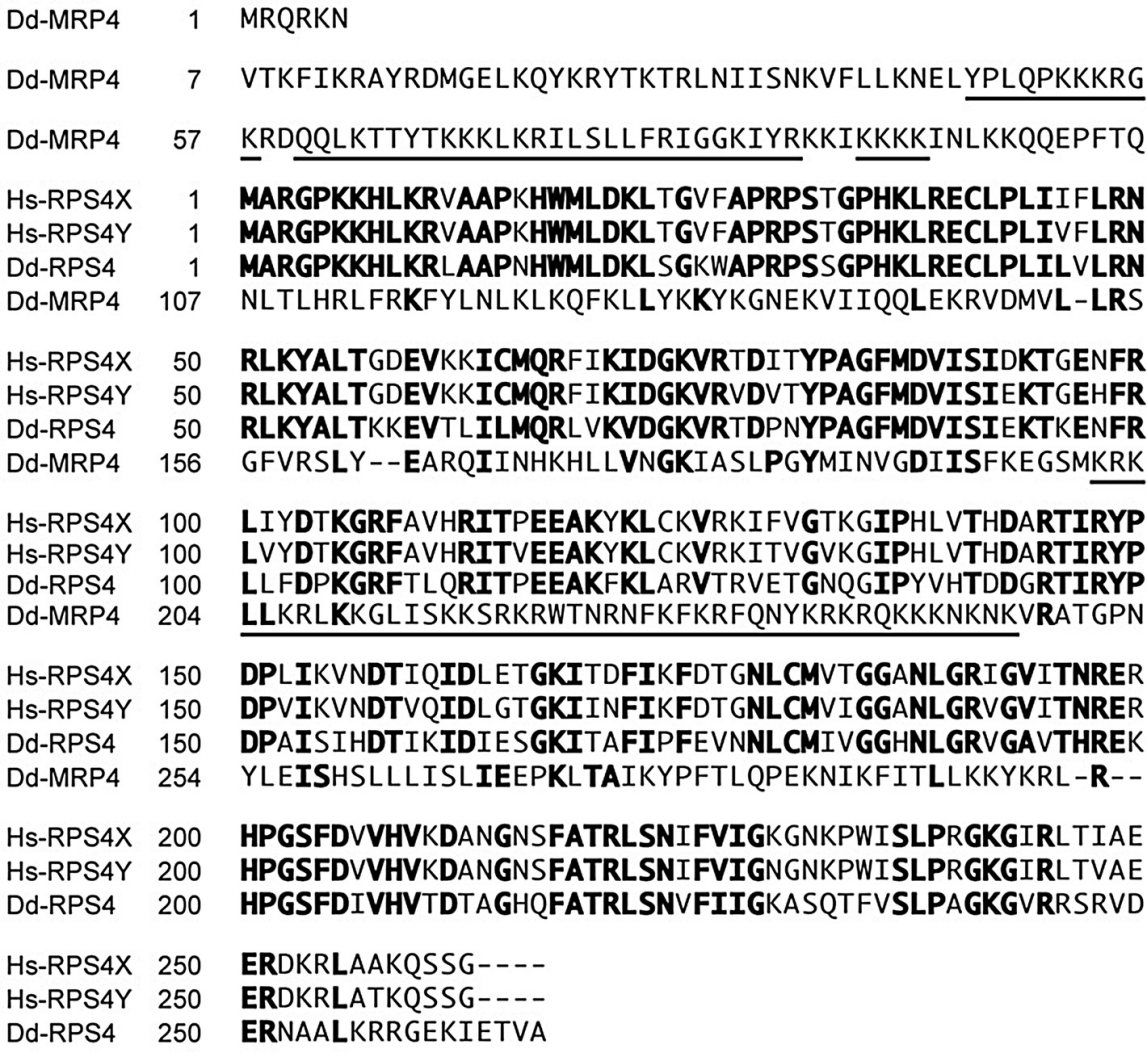
**Alignment of *Dictyostelium *MRP4 (Dd-MRP4), human RPS4X (Hs-RPS4X), human RPS4Y (Hs-RPS4Y), and *Dictyostelium *RPS4 (Dd-RPS4).** Amino acid sequences were deduced from full-length of cDNAs. Spaces (hyphens) indicate gaps proposed to optimize alignment; block letters, matching residue among either Hs-RPS4X, Hs-RPS4Y, and Dd-RPS4 or between Dd-RPS4 and Dd-MRP4; predicted nuclear localization sequences in Dd-MRP4 are underlined.

The human homologue of mitochondrial Dd-MRP48 (300 amino acids) is probably RPS4X (Hs-RPS4X; 263 amino acids) that is encoded by a nuclear gene of the human sex chromosome X and an isoform of ribosomal protein S4 [15,26]. RPS4Y (Hs-RPS4Y; 263 amino acids) encoded by the human sex chromosome Y has an amino acid sequence similar to Hs-RPS4X, and differs only at 19 of 263 amino acids (Figure 2) [27]. Somewhat surprisingly, though *Dictyostelium* is evolutionally far from human, the homology of Dd-RPS4 (267 amino acids) and Hs-RPS4X, both of which is present as a subunit of cytoplasmic ribosomes but not in mitochondria, is considerably high (66% Identity, 92% Similarity in the amino acid sequence), as shown in Figure 2. In contrast, Dd-MRP4 has less similarity (38% Identity, 76% Similarity in the amino acid sequence) to RPS4X (Hs-RPS4). This seems to indicate that the *Dd-mrp4* gene and its product (Dd-MRP4) were persistently retained in mitochondria without being transferred to the nucleus during the course of evolution. Here it is of interest to note that Dd-MRP4 is very unique in that it has several nuclear localization signals within the molecule (Figure 2; underlined parts). Also, the reason why Dd-MRP4 can be encoded by mitochondrial genome itself in the cytoplasm of *Dictyostelium* cells is presently unknown and remains to be elucidated as a chain of evolutionally amazing and rather unexpected incident, because MRPs are generally encoded by nuclear genome in eukaryotic cells.

Fortunately, judging from the Codon Usage Database (NCBI-GenBank), it was found that the amino acid sequence of Dd-MRP4 expressed enforcedly by the pcDNA3.1-*mrp4* vector in the cytoplasm of human cells is completely the same as that of Dd-MRP4 naturally expressed in *Dictyostelium* mitochondria (data not shown). In order to allow the Dd-MRP4 expression in human mitochondria, we tried to make a vector construct bearing a mitochondrial localization signal (MLS), but strangely failed to obtain any *Escherichia coli* clone that could express Dd-MRP4 as well as Dd-MRP4 with MLS at the N-terminus in spite of at least 20 times of trials, because of unknown reasons.

### Specific suppression of the proliferation of human tumor cells by the ectopically enforced expression of *Dd-mrp4* gene

**Figure 3:**
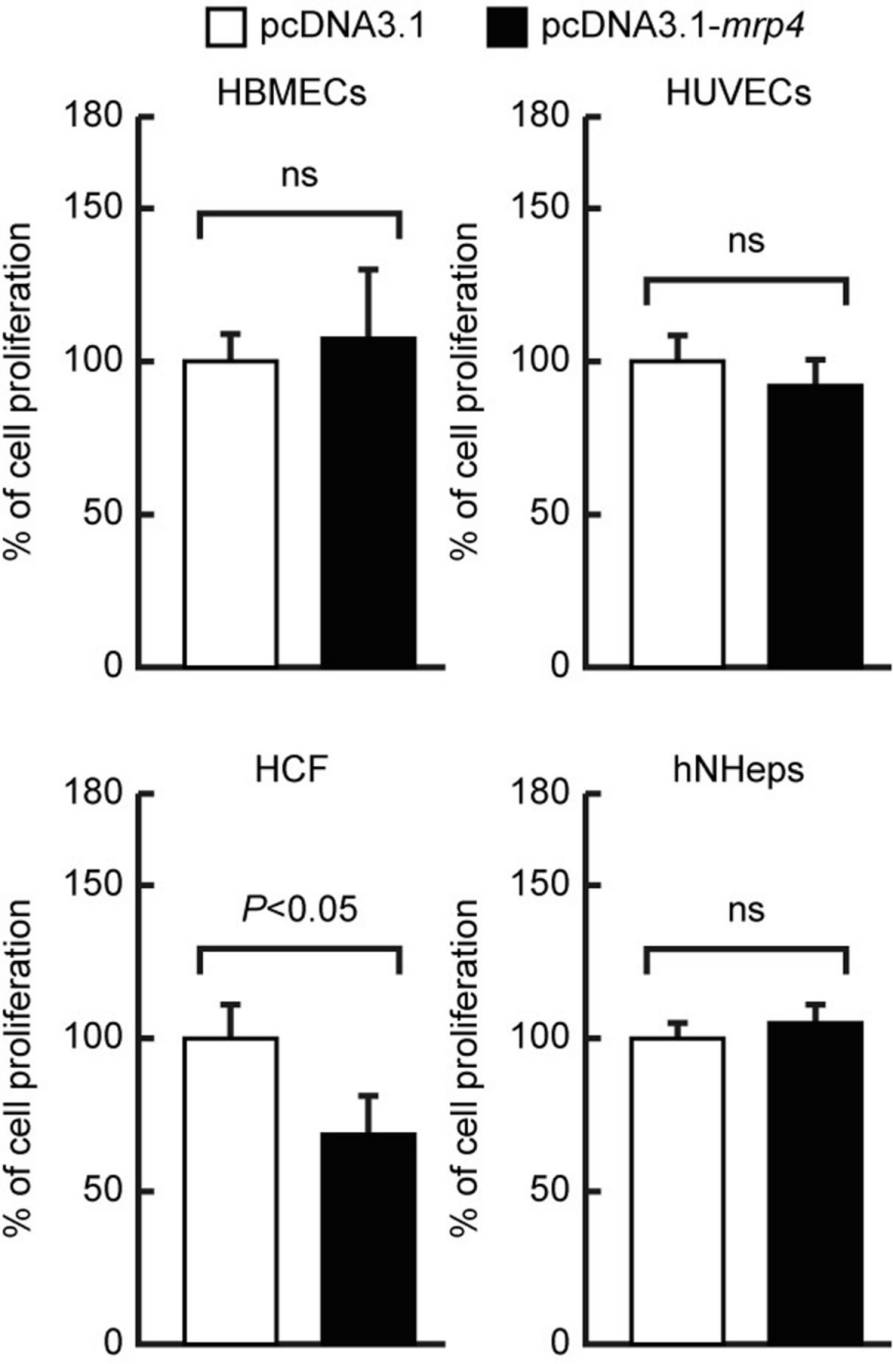
**Effects of enforced *Dd-mrp4 *expression on the proliferation of several human primary cultured cells.** HBMECs, HUVECs, HCV and hNHeps cells were seeded in a 6-well plate and transfected with the pcDNA3.1, pcDNA3.1-*rps4* (negative control), or pcDNA3.1-*mrp4* vector for 6 hrs. After 42 hrs of incubation at 37°C, the cells were treated with WST-8 solution for 2 hrs, and their viability was monitored as described in Methods. The results represent the mean ± SD in three independent experiments. ns; not significant.

To know if the ectopically enforced expression of *Dd-mrp4* gene is effective on growth of the primary cultured cells, such as human brain microvascular endothelial cells (HBMECs) and human umbilical vein endothelial cells (HUVECs), they were transfected with three kinds of vector constructs (pcDNA3.1 (Mock), pcDNA3.1-*rps4*, or pcDNA3.1-*mrp4* vector), and this was followed by incubation in growth medium. As a result, no significant effects on cellular proliferation were observed with one exception (human cardiac fibroblasts: HCF), as shown in Figure 3. human cardiac fibroblasts (HCF) were originally isolated from the ventricles of an adult heart. They are known to play a central role in the maintenance of the extracellular matrix in the normal heart and the synthesis of growth factors and cytokines [28]. Under pathophysiological conditions, HCFs are involved in restoration of a scar after cardiac fibrosis, and cardiac hypertrophy [28]. Although the reason why the HCF growth was rather suppressed by the enforced expression of *Dd-mrp4* gene is presently unknown, it is possible that HCFs might have a somewhat tumor-like nature.

**Figure 4:**
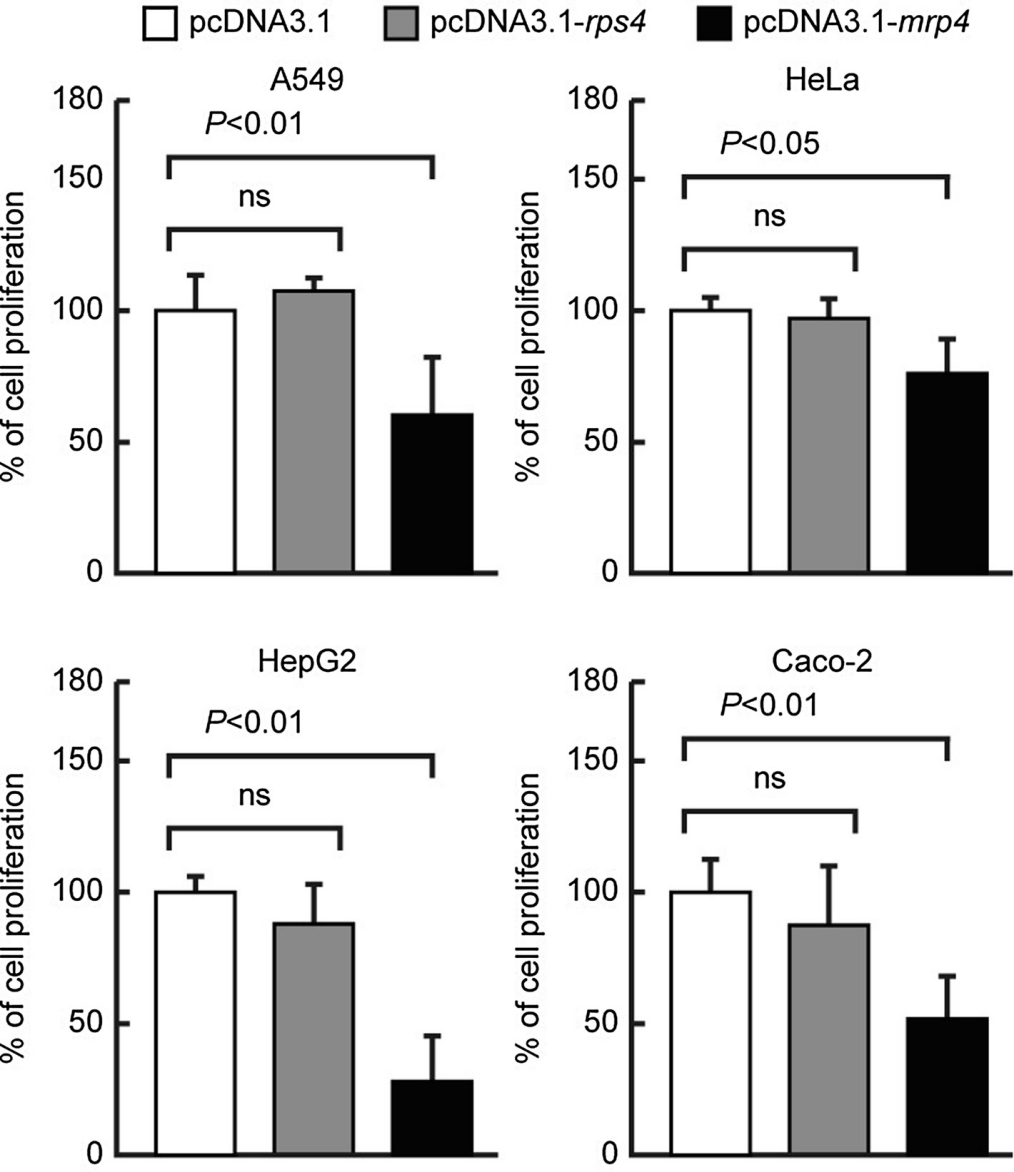
**Inhibitory effects of enforced *Dd-mrp4 *expression on the proliferation of several human tumor cell lines.** Tumor cell lines (A549, HeLa, HepG2, Caco-2 cells) were seeded in a 6-well plate and transfected with the pcDNA3.1, pcDNA3.1-*rps4* (negative control*)*, or pcDNA3.1-*mrp4* vector for 6 hrs at 37°C. After 48 hrs of incubation at 37°C, the cell viability was measured as described in Methods. The results represent the mean ± SD in three independent experiments. ns; not significant.

Importantly, it is of value to note that enforced *Dd-mrp4* expression is capable of suppressing significantly growth in all of the tumor cell lines (Caco-2, A549, HeLa, and HepG2 cells), though the levels of growth suppression is somewhat different depending on the tumor cell lines used: the most remarkable suppression of growth was noticed in HepG2 cells (Figure 4). This indicates that the *Dd-mrp4* mRNA and/or Dd-MRP4 protein have a potent suppressive effect on the proliferation of at least several human tumor cell lines.

**Figure 5:**
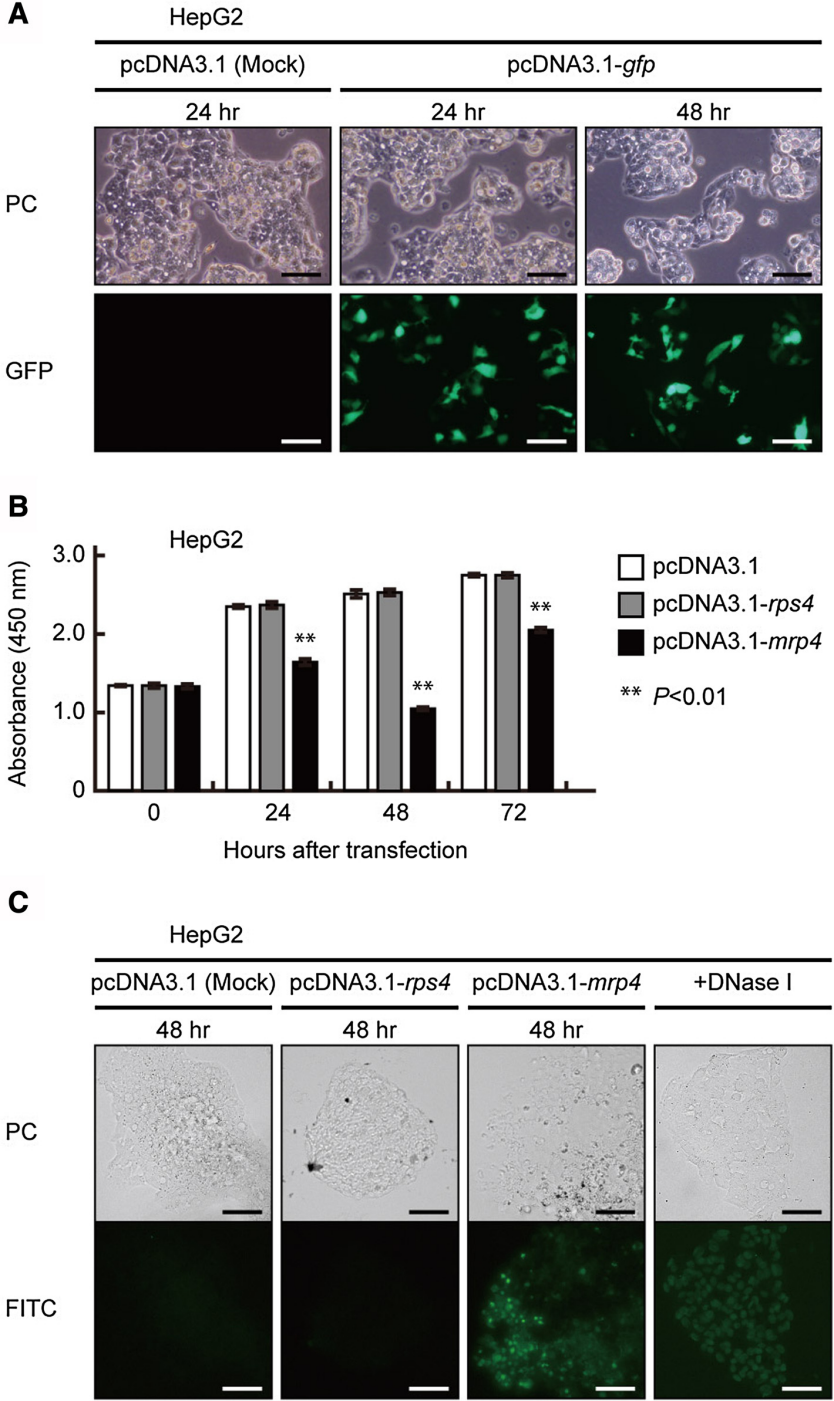
**Growth kinetics and morphological characteristics of human tumor HepG2 cells trnsnfected with the pcDNA3.1, pcDNA3.1-*rps4 *(negative control), or pcDNA3.1-*mrp4 *vector construct. (A)** Efficiency of vector transfection into HepG2 cells. At 24 and 48 hrs after transfection of the pcDNA3.1 (Mock) or pcDNA3.1-*gfp* (inserted instead of *rps4*), phase-contract (PC) and fluorescence (GFP) micrographs were taken to evaluate the efficiency of vector transfection. It is manifest that a considerable number of cells are strongly stained with GFP. Bar, 20 μm. **(B)** After the vector transfection, HepG2 cells transfected by the pcDNA3.1 (Mock) or pcDNA3.1-*rps4* (negative control) continue to increase their cell number in growth medium. In the case of HepG2 cells transfected by the pcDNA3.1-*mrp4*, increase in the cell number is significantly suppressed during the first 48 hrs of incubation. However, once the decreased number of cells begins to increase during 48-72 hrs after transfection. The results represent the mean±SD in three independent experiments. **(C)** TUNEL assay. HepG2 cells transfected with the pcDNA3.1 (Mock), pcDNA3.1- *rps4*, or pcDNA3.1-*mrp4* were incubated for 48 hrs and subjected to a TUNEL assay, as described in Methods. From morphological assessment of apoptosis detected by FITC-staining (FITC) and phase-contrast (PC) images of the same field, it is evident that HepG2 cells transfected with the pcDNA3.1 (Mock) or pcDNA3.1- *rps4* are not stained with the FIFC-conjugated antibody, but that HepG2 cells ectopically expressing the pcDNA3.1*-mrp4* are strongly stained with the antibody. As shown in the most-right column, when HepG2 cells transfected with the pcDNA3.1 (Mock) was pretreated with DNase-I, they were stained with the FITC-conjugated antibody because of DNA fragmentation as observed in the process of apoptosis. Bar, 50 μm.

Figure 5 shows growth kinetics and morphological characters of HepG2 cells transfected by the several vector constructs. The efficiency of vector transfection was monitored using HepG2 cells transformed by pcDNA3.1-*gfp* vector. Judging from cell counts of GFP-stained cells, 37.9 ± 2.8% of cells were estimated to be transformed by the vector, though the degree (i.e. the strength of GFP fluorescence in cells) of transfection was considerably differential in the cell population (Figure 5A).

When HepG2 cells transfected with the pcDNA3.1 (Mock) or pcDNA3.1-*rps4* were incubated in growth medium, they continued to proliferate at least during the first 72 hrs of incubation after the vector transfection (Figure 5B). In the case of HepG2 cells transfected with the pcDNA3.1-*mrp4* vector, however, their proliferation was fairly repressed during the first 24 hrs of incubation, and the cell number was rather decreased during another 24 hrs of incubation, as shown Figure 5B. Here it is notable that the once the decreased cell number increases during another 24 hrs of incubation (Figure 5B). Although the precise reason for this increase of cell number is presently unknown, it is quite possible that HepG2 cells with little or no *Dd-mrp4* gene, i.e. scarcely transformed cells in the population, may grow and multiply.

To know if the growth suppression in HepG2 cells transfected with the pcDNA3.1-*mrp4* vector is due to apoptotic cell death, we performed a TUNEL assay. As a result, HepG2 cells transfected with the pcDNA3.1 (Mock) or pcDNA3.1-*rps4* were not stained with the FIFC-conjugated antibody, while that HepG2 cells expressing the pcDNA3.1*-mrp4* were destined to die by apoptosis and therefore were strongly stained with the antibody because of DNA fragmentation (Figure 5C). Incidentally, when HepG2 cells transfected with the pcDNA3.1 (Mock) were pretreated with DNase-I, they exhibited FITC-staining (the most-right column of Figure 5C). Only, in the population of HepG2 cells tried to be transfected with the pcDNA3.1-*mrp4* there were observed a considerable number cells that was not stained with the FITC-conjugated antibody. Judging from the transfection efficiency (presumably ca. 38% in the case of HepG2 cells) described above, they would be most probably HepG2 cells that were failed to be transfected with the pcDNA3.1-*mrp4*.

## Discussion

In eukaryotic cells, mitochondria are self-reproducing organelles with their own DNA and they play a central role in ATP synthesis by respiration. Increasing evidence indicates that mitochondria also have critical and multiple functions in the initiation of cell differentiation, cell-type determination, cell movement, and pattern formation. This has been most strikingly realized in development of an excellent model organism, *Dictyostelium discoideum*[20]. For example, the expression of *Dd*-*mrp4* gene is essential for the initiation of cell differentiation, as previously described [20-22]. The *Dictyostelium* homologue (Dd-TRAP1) of TRAP-1 (tumor necrosis receptor-associated protein 1), a mitochondrial molecular chaperone belonging to the Hsp90 family, allows the pecocious transition of cells from growth to differentiation via a novel prestarvation factor (PSF-3) in growth medium [28,29]. Moreover, a cell-type-specific organelle named a prespore-specific vacuole (PSV) is constructed by mitochondrial transformation with the help of the Golgi complex [21,30,31].

In *Dictyostelium*, mitochondrial large ribosomal RNA (mtlrRNA) is required for normal phototaxis and thermotaxis of a migrating pseudoplasmodium (slug) [32]. It has been shown that the mitochondrial function is also impaired by mutations affecting nuclear-encoded proteins required for correct folding in the organelle of both mitochondrially- and nuclear-encoded proteins, and that antisense RNA inhibition of the expression of chaperonin 60, one of such proteins, impairs signal transduction for phototaxis of *Dictyostelium* slugs [33]. With respect to chemotaxis, a novel mitochondrial protein (Tortoise) has been shown to be essential for directional movement of *Dictyostelium* cells in cAMP gradients [34]. The *Dictyostrelium* mitochondria are also closely involved in a variety of cellular activities including CN-resistant respiration and apoptosis.

As reported previously, transformants (*Dd-mrp4*^AS^ cells) generated by antisense-mediated gene inactivation exhibit markedly delayed differentiation, while the initial step of cell differentiation is enhanced in *Dd-mrp4*^OE^ cells overexpressing the *Dd*-*mrp4* mRNA in the extramitochondrial cytoplasm [21]. Here it is of interest to note that the antisense-*mrp4* RNA synthesized in the extramitochondrial cytoplasm is effective as the partial disruption of *Dd-mrp4* gene. This seems to indicate that a trace of the *Dd-mrp4* mRNA and/or Dd-MRP4 protein, both of which are synthesized in mitochondria, may be released to the extramitochondrial cytoplasm. Alternatively, it is also possible that the antisense-*mrp4* RNA may enter mitochondria to inactivate *Dd*-*mrp4* mRNA. Based on PSORTII prediction, mysteriously enough, the Dd-MRP4 is very unique in that it is a mitochondrial protein encoded by mt-DNA itself, but has several nuclear localization signals in the molecule [21]. Actually, it has been confirmed that the Dd-MRP4 protein produced in the cytoplasm of the *Dd-mrp4*^OE^ cells is preferentially transferred into the nucleus [35]. Although the fact that only the MRP4 protein of *Dictyostelium* has several nuclear localization signals is puzzling, at least a part of the Dd-MRP4 protein seems to work in the nucleus to regulate cell differentiation. In other organisms, their MRP4 proteins have no nuclear localization signals. It is generally difficult for proteins located in the mitochondrial matrix to go out to the cytosol, because mitochondria are partitioned by two (outer and inner) membranes. However, several mitochondrial proteins like apoptosis-inducing factor (AIF; [36]), endonuclease G (EndoG; [37]), and heat shock protein 70 (Hsp70; [38]) have been shown to move to the nucleus in response to apoptosis and heat shock. All of these proteins are encoded by the nuclear genome DNA, followed by translocation to the mitochondrion and then again to the nucleus. Thus the behavior of *Dictyostelium* MRP4 produced from the mitochondrial genome DNA must be greatly notable, though the mechanism by which the ectopically expressed *Dd-mrp4* in human tumor cells is capable of suppressing specifically their proliferation is presently unknown and remains to be elucidated in future studies. Provided that it is possible to deliver Dd-MRP4 with a MLS at the N-terminus into human mitochondria by the use of a well-directed vector construct, one might expect a more marked inhibitory effect of Dd-MRP4 on tumor growth, as compared with Dd-MRP4 lacking a MLS. Recently, importance of human ribosomal protein S4 (RPS4X) produced from the nuclear genome DNA (sex-chromosome X) has been regarded as a predictive and prognostic marker in human serous epithelial ovarian cancer, because its high expression is coupled with a lower risk of individual death and later disease progression, as compared to low expression of RPSX4 [15]. This seems to indicate that RPSX4 might restrain the progression of aggressive cancers, possibly through preferential suppression of tumor proliferation by means of their selective apoptosis, as in the case of Dd-MRP4.

Apoptosis is a physiological cell suicide program that is critical for the normal development and maintenance of healthy tissues. Inhibition of apoptosis brings numerous cancers, autoimmune diseases, inflammatory diseases, and viral infections, because cells are excessively accumulated by an increase of cellular proliferation. The cancer is often characterized by an overexpression of IAP (Inhibitor of Apoptosis) family members. The best characterized IAP is XIAP (X-linked inhibitor of apoptosis protein), which binds caspase-9, caspase-3 and caspase 7, thereby inhibiting their activation and preventing apoptosis. As a result, the malignant tumor cells undergo an abnormal response to apoptosis induction: cell-cycle regulating genes (such as *p53*, *ras* or *c-myc*) are mutated or inactivated in diseased cells, and further genes such as *Bcl-2* also modify their expression in tumors. For example, p53 prevents the cell from replicating by arresting the cell cycle at G1 to give the cell time to repair the DNA-damage, but it induces apoptosis if the damage is too extensive to repair [39]. Therefore, any disruption to the regulation of the p53 or interferon genes results in impaired apoptosis, followed by the possible formation of tumors. In this connection, it has been demonstrated that p53 and E2F are crucial for the induction of cell death downstream from retinoblastoma suppressor (RB) deficiency [40-45]. Recently, Hilgendorf et al. [7] have shown that a fraction of the RB pool localizes constitutively to the mitochondria, where Bax (Bcl-2-associated X protein) is normally resided in IMR human cells and mouse liver cells, including phosphorylated forms of RB, and that RB is able to interact with Bax, conformationally activate it, and then trigger mitochondrial outer membrane permeabilization (MOMP), followed by cytochrome *c* release, as an essential step in the initiation of apoptosis. Importantly, an RB mutant lacking the nuclear localization signal but carrying a mitochondrial import signal has been shown to be enough to drive apoptosis [45]. Such remarkable ability of RB is also noticed in *p53*-null oestrosarcoma tumor cells [46]. Taken together these data suggest that the proapoptotic action of RB at the mitochondria may be essential as its transcriptional tumor suppressor function, and further this RB function is p53-independent. On the other hand, it has been found that p53 interacts with Bcl-2 or Bcl-X_L_ at the mitochondria to neutralize their activity and thus activate proapoptotic Bax (Bcl-2-associated X protein) and Bak (BRI1-associated receptor kinase) proteins to drive MOMP. This has been confirmed by the fact that tumor-derived *p53*-null mutants fail to interact with Bcl-2 or Bcl-X_L_[47]. Recently, the role of p53 has been shown to be to extend even beyond apoptosis; p53 can enter the mitochondrial matrix and then drive necrosis [48].

## Conclusion

The most important finding made by the present work is the fact that the ectopically enforced *Dd-mrp4* expression can suppress specifically all of the tumor cell lines examined, but not most of the primary cultured cells. Although the action mechanism in apoptosis-related signaling pathways are presently unknown, it is most likely that *Dd-mrp4* mRNA and/or Dd-MRP4 protein expressed extraneously in human tumor cells may carry out the p53- or RB-like functions to suppress their proliferation via the induction of proapoptosis. This also suggests that the *Dd-mrp4* gene derived from *Dictyostelium* mitochondria may provide a new promising therapeutic strategy for disrupting cell viability pathways in human cancers. In this connection, the establishment of an improved method for transfecting human cancer cells more efficiently with the *Dd-mrp4* gene will serve to induce more completely the apoptotic cell death of human tumors.

## Abbreviations

GDT: Growth/differentiation transition; RPS4: Ribosomal protein S4; MRP4: Mitochondrial ribosomal protein S4; Dd-MRP4: Mitochondrial ribosomal protein S4 of *Dictyostelium discoideum*; Bcl-2: B-cell CLL/lymphoma 2; MAPK: Mitogen-activated protein kinase; JNK: c-jun N- terminal kinase; DAP3: Death-associated protein 3; PDCD9: Programmed cell death protein 9; MRPL41: Mitochondrial ribosomal protein L41; RPS29: S29 ribosomal protein; TRAP-1: Tumor necrosis receptor-associated protein 1; Hsp70: Heat shock protein 70; ATP: Adenosine triphosphate; cAMP: 3′, 5′-cyclic adenosine monophosphate; MidA: Mitochondrial dysfunction protein A; DUF: Domain of unknown function proteins; AIF: Apoptosis-inducing factor; EndG: Endonuclease G; IAP: Inhibitor of Apoptosis; XIAP: X-linked Inhibitor of Apoptosis protein; MOMP: Mitochondrial outer membrane permeabilization; RB: Retinoblastoma protein; P53: Phosphoprotein p53 (tumor suppressor p53); *ras*: *rat sarcoma* gene; c-myc: retroviral v-*myc* oncogene; Bax: Bcl-2-associated X protein; Bak: BRI1-associated receptor kinase; E2F: E2F transcription factor.

## Competing interests

The authors declare that there are no conflicts of interests.

## Authors’ contributions

Conceived and designed the experiments: JC YM. Performed the experiments: JC HA. Analyzed the data: JC YM. Wrote the paper: JC YM. All authors read and approved the manuscript.
